# An Experimental Method for Measuring Mechanical Properties of Rat Pulmonary Arteries Verified With Latex

**DOI:** 10.6028/jres.108.018

**Published:** 2003-06-01

**Authors:** E. S. Drexler, A. J. Slifka, J. E. Wright, C. N. McCowan, D. S. Finch, T. P. Quinn, J. D. McColskey, D. D. Ivy, R. Shandas

**Affiliations:** National Institute of Standards and Technology, Boulder, CO 80305; Department of Pediatrics, Division of Cardiology, University of Colorado, Health Sciences Center, Denver, CO 80218; Department of Pediatrics, Division of Cardiology, University of Colorado, Health Sciences Center, Denver, CO 80218Department of Mechanical Engineering, University of Colorado, Boulder, CO 80309

**Keywords:** apparatus, biaxial test, latex, mechanical properties, nonlinear material, pulmonary hypertension, rat pulmonary artery

## Abstract

This paper describes a test method for measuring the mechanical properties of small, nonlinear membrane samples from a rat model for pulmonary hypertension. The size and nonlinearity of the pulmonary artery samples poses a challenge for developing a test method that will generate quality, reproducible data in the pressure range experienced by the hypertensive pulmonary artery. The experimental method described here has sufficient precision to yield a combined relative standard uncertainty of 4 %. The method is calibrated against 75 µm thick latex and the data agree well with the neo-Hookian model.

## 1. Introduction

Pulmonary hypertension is an important factor in determining post-operative morbidity and mortality in children with congenital heart disease. The additional right-side afterload and the relative inability of the right ventricle to cope with chronic volume and pressure loading can also lead to catastrophic failure of the right heart if left untreated. The etiology of the disease is still not completely understood, although two general distinctions are made: primary and secondary. Primary pulmonary hypertension is a diagnosis of exclusion, where no single causative parameter has been identified; secondary pulmonary hypertension can occur due to a number of reasons, including the congenital heart defect itself. Regardless of the underlying manifestation, pulmonary hypertension in children continues to be a significant clinical diagnosis, especially for children born at altitudes appreciably above sea level.

Several novel pharmaceutical agents are being developed for pre-operative and chronic treatment of pediatric pulmonary hypertension. These include prostaglandin analogs such as prostacyclin and low-dose inhaled nitric oxide. Clinical studies [1] have shown that these agents lower pulmonary vascular resistance and therefore appear promising in treating pulmonary hypertension. However, very little is known about the biomechanics and hemodynamics of pediatric pulmonary hypertension. Such information would: allow development of novel non-invasive methods to evaluate pulmonary hypertension (the current standard uses invasive catheterization techniques); provide fundamental information about the dynamics of the reactive vasculature associated with pulmonary hypertension; facilitate development of numerical models incorporating blood flow and arterial wall characteristics, which in turn can be used to study disease progression, reaction to clinical interventions, long-term changes, etc.; provide information on key metrics that may be better markers of clinical outcome than pulmonary vascular resistance.

Current research includes developing non-invasive markers of pulmonary vascular resistance, numerical modeling of right-sided fluid dynamics, understanding pulmonary artery mechanics in vivo using intravascular ultrasound techniques, and developing new markers of pulmonary reactivity [2–5]. One missing link, however, is an understanding of the fundamental biomechanics of the pulmonary arteries in pulmonary hypertension. Furthermore, very little information is available in the literature on this topic. In this regard, the availability of animal models of inflammatory and restrictive pulmonary hypertension presents a good opportunity to study the biomechanics of pulmonary hypertension. The Sprague-Dawley rat model, with pulmonary hypertension induced through hypoxia or injection of monocrotaline, in particular, has been extensively used for pulmonary hypertension studies. The proximal pulmonary arteries from these animals can be easily harvested for biomechanical testing. However, the extremely small size of these arteries presents unique experimental issues for mechanical testing. This paper represents our initial efforts in the development and validation of the experimental apparatus to mount and test the main pulmonary arteries from the rat.

**Fig. 1:**
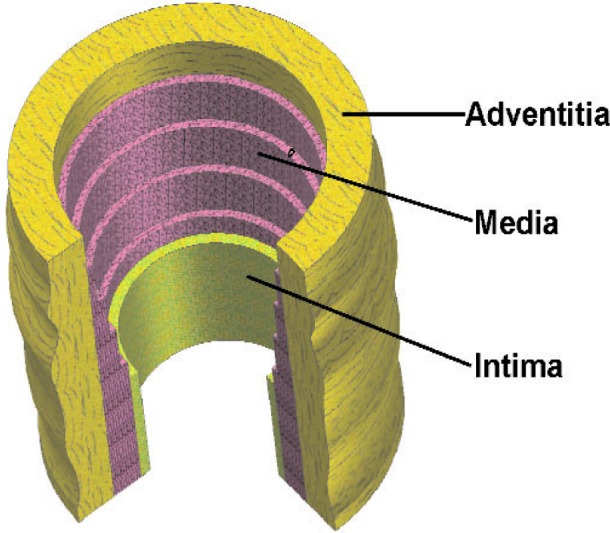
Diagram showing the components of the arterial wall.

Physiologically there are two broad categories of arteries, elastic and muscular. Elastic arteries, such as the pulmonary artery, have a ratio of collagen to smooth muscle cells that is larger than that observed in muscular arteries. Three layers comprise the arterial wall: the intima, the media, and the adventitia (see Fig. 1). Endothelial cells line the intima layer, and it is believed that there is minimal remodeling (restructuring) of the intima during the development of pulmonary hypertension. The media contains collagen fibers, smooth muscle cells, and elastin in a three-dimensional layered structure. The gross structure appears as smooth muscle cells, circumferentially oriented, surrounded by elastin with collagen fibers in between. Smooth muscle cells are known to remodel with hypertension, and drug therapies used at present are directed toward that remodeling. Within the adventitia is a mixture of elastin and collagen that has a helical formation and is nearly axially oriented. The adventitia is a passive layer, and the role of remodeling within this layer has not been previously studied. During hypertension the pulmonary artery experiences minimal pressures on the order of 3.3 × 10^3^ Pa (25 mm Hg), and maximal pressures as high as 20 × 10^3^ Pa (150 mm Hg).

## 2. Approach

In order to characterize the progression and effects of the disease, a study of rats was initiated. Rats were chosen for their rapid maturation, for the ease of chemically inducing pulmonary hypertension, and because of the facility of obtaining a genetically identical population. One component of the rat study is to measure and compare the mechanical properties of healthy and diseased arterial tissue. The size of the rats and the difficulty in excising the pulmonary artery introduce some challenges in measuring the mechanical properties. The dimensions of the main pulmonary arteries when we receive them are approximately 2.5 mm in diameter by 4 mm long, so it was necessary to design a test system that can generate meaningful, reproducible data. It was also essential that the data contribute toward a constitutive model of the arteries in their healthy and diseased conditions.

A review of the literature showed that there are numerous test methods and constitutive models [6–13] for testing and characterizing tissue. It is also clear from the literature [6,7,10] that the anisotropic nature of arteries cannot be properly addressed by uniaxial tensile tests. Reference [6] offers a comprehensive review of the test methods commonly employed, and methods used by other researchers testing rat arteries [14–16] were considered. Combining tension, inflation, and torsion in a single test provides the comprehensive representation of material behavior, but a good test to approximate the functionality of the hypertensive pulmonary artery is the bubble test. Particularly if inversion of the artery is incorporated into the testing procedure, as suggested in [6], it may be possible to obtain the complete set of constants needed to characterize the material behavior. The test will allow for the anisotropic displacements anticipated in the longitudinal and circumferential directions of the artery without decoupling them. This manuscript will describe the design of the test system and an inflation test conducted on latex sheet used to validate the system.

With the many test techniques described in the literature, data can be directly compared only through a constitutive model. It was, therefore, necessary for us to choose a well-conceived constitutive model for which we can acquire and fit the requisite data. A material that exhibits nonlinear strain behavior and large deformations at small applied stresses is not adequately characterized by Hooke’s law, and a functional expression is needed to describe its stress-strain relationship. Such a material may be defined by the strain energy function, which relates the internal energy contained in the strained material to its three-dimensional strain. The strain energy can be defined as the mechanical work required in a reversible process to produce a particular state of stress [17]. The strain energy function is the most comprehensive approach for these types of materials and can characterize their behavior from small deformations up to bursting. Models based on a computational expression for the strain energy function of thick-walled tubes are described in Refs. [6,10]. Reference [10] reviews five two- and three-dimensional constitutive models that have been published in the literature, as well as the author’s proposed model, which accounts for the different properties of the multi-layered arterial wall:

$$\Psi_{M,A}=\frac{c_{M,A}}{2}\left(I_{1}-3\right)+\frac{k_{1M,A}}{2k_{2M,A}}\sum_{i=4,6}\left\{\exp\left[k_{2M,A}{\left(I_{M,A}-1\right)}^{2}\right]-1\right\},$$ (1)

where *Ψ* is the strain energy function for either the medial (M) or adventitial (A) layers, *I*_i_ represents the invariants, and *c* and *k* are the material parameters. The first term in Eq. (1) is associated with isotropic deformations, whereas the second term is associated with anisotropic deformations of the arterial wall. For the analysis of latex that will be presented here, the above strain energy function simplifies to a single-layer model with only the isotropic term

$$\Psi =\frac{c}{2}\left(I_{1}-3\right).$$ (2)

Note that Eq. (2) represents the neo-Hookean model, often used for rubber-like materials, and is also the same form that was presented in Refs. [8,18].

## 3. Test Procedure

**Fig. 2:**
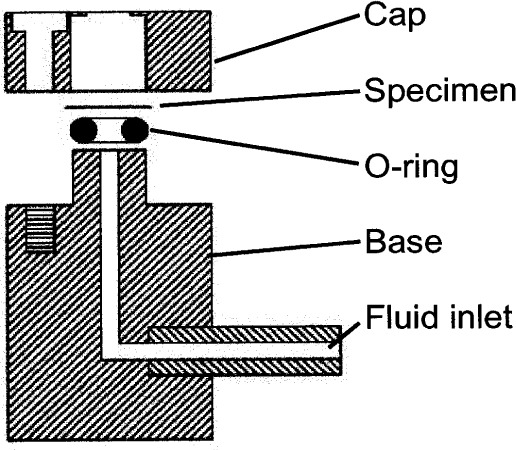
Sectional view of the biaxial, bubble test fixture.

**Fig. 3:**
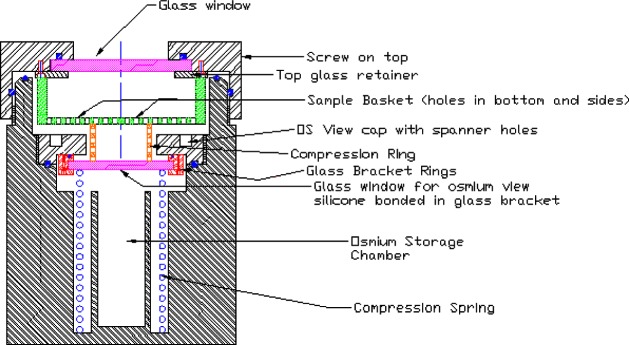
Sectional view of the Os staining chamber.

**Fig. 4:**
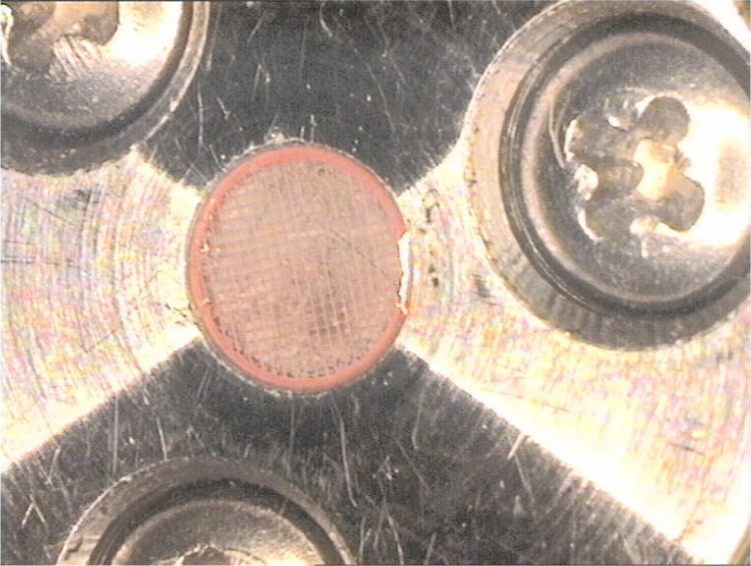
Image showing the TEM grid placed over the latex memberane within the bubble test fixture prior to Os staining.

Our experimental setup is similar to many of those described in the literature [8,18–22], and more recently in [23]. References [19] and [23] suggest the suitability of the bubble inflation test for membrane-like soft tissues. A material may be treated as a membrane if the ratio of the thickness to the principal radius of curvature is less than about 0.1 [24], which we fully expect with the present test fixture. Qualitatively, the inability of the rat’s pulmonary artery to support out-of-plane shear is indicative of membrane-like behavior. As with the system described in [23], our fixture is designed for small specimens. Figure 2 shows a sectional view of our modified bubble test fixture; the stainless steel fixture has an aperture of 2.78 mm. In advance of testing tissue, latex was used to test the performance of the fixture and experimental technique. The latex sample was stained in a square array with OsO_4_ vapor [25] using a copper grid designed for a transmission electron microscope (TEM) with a 74 µm pitch for the mask; a sectional view of the OsO4 chamber is shown in Fig. 3. Whereas it is understood that the use of OsO_4_ introduces chemical cross-links, the exposure times were kept sufficiently short to allow only the surface to be stained. The latex was placed within the biaxial fixture, the grid clamped beneath the cap, and the sample pressurized just enough to prevent the OsO_4_ from leaking around the mesh. The entire fixture was placed in the chamber of Fig. 3 and exposed to the OsO_4_ for 2 min, during which the chamber remained under slight positive pressure; then the grid was removed before testing. Figure 4 shows an image of the latex sample with the TEM grid affixed to its surface.

**Fig. 5:**
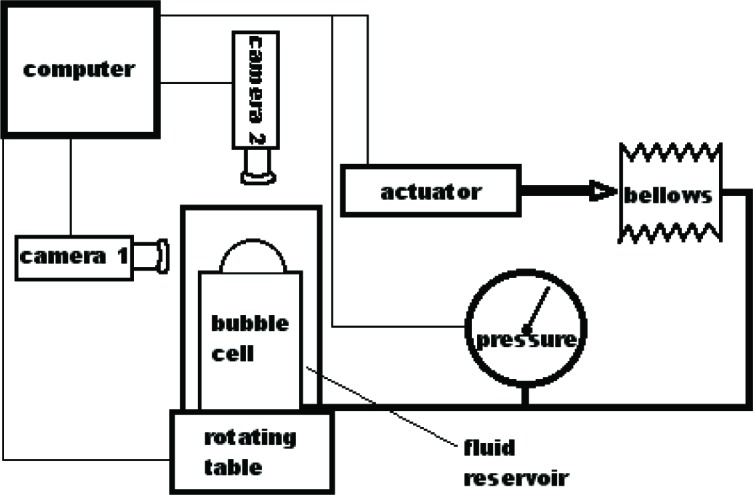
Schematic of the computer-controlled test system.

The experiment is designed so that each component that contributes to the data set has computer control (Fig. 5), similar to that in the experimental design in [23]. Through a computer program, the motion controller can move the bellows with feedback from the pressure transducer, so that pressure can be applied and recorded in well-controlled increments (± 13.8 Pa). Upon reaching the predetermined pressure setting, the multi-channel video control card allows images to be collected from camera 1 at 30° intervals, controlled by the computer-driven rotating stage, from 0° to 150°, and from camera 2 along the pole direction. The OsO_4_ pattern on the specimen is readily visible in the images collected by camera 2 and will be used to define the curved surface of the anisotropic tissue. Collecting images from multiple orientations will permit flexibility in visualizing and in analyzing the deformation. Once the experiment has been set up, pressurization and data collection are fully automated, with refocusing being the only reason for operator interruption.

The specimen-containing fixture sits in a reservoir (see Fig. 5) of de-ionized water for the latex test, or in a buffered saline solution with a recirculating bath set to 37 °C for testing the pulmonary arteries. De-ionized water or buffered saline solution for latex or tissue, respectively, fills the bellows and tubing that comprise the pressure system, so that neither surface of the specimen is in contact with air during testing.

## 4. Experiment

**Fig. 6:**
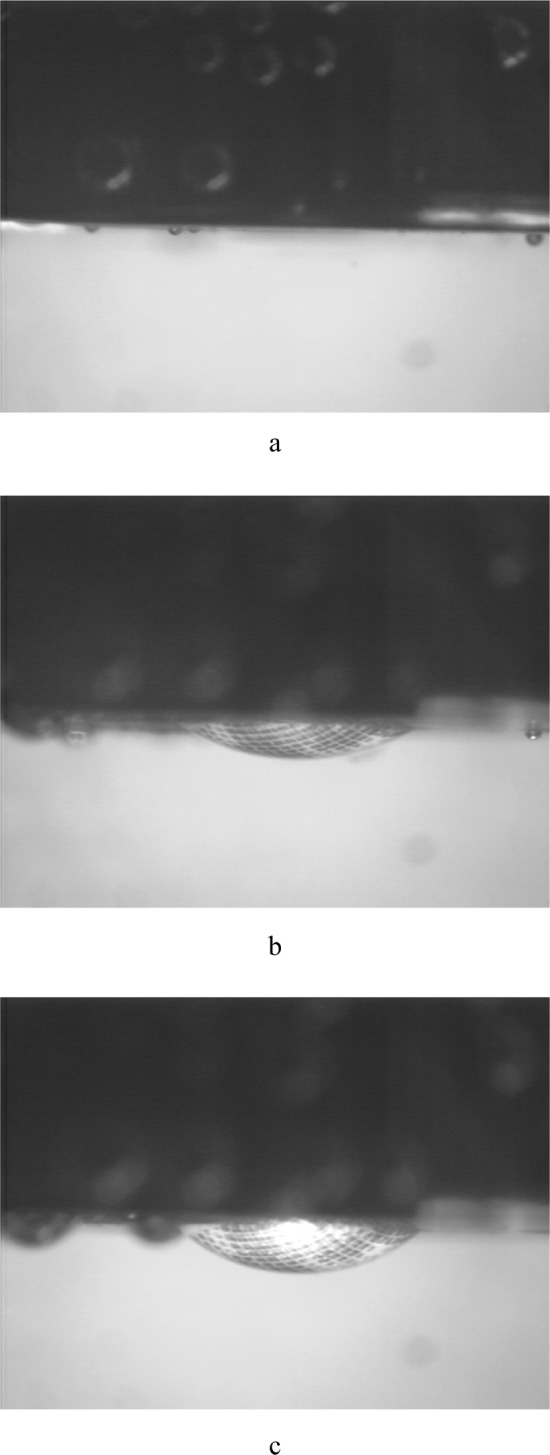
Digital images of the profile from the latex bubble test collected at (a) 0 Pa, (b) 6.90 × 10^3^ Pa, and (c) 1.38 × 10^4^ Pa.

**Fig. 7:**
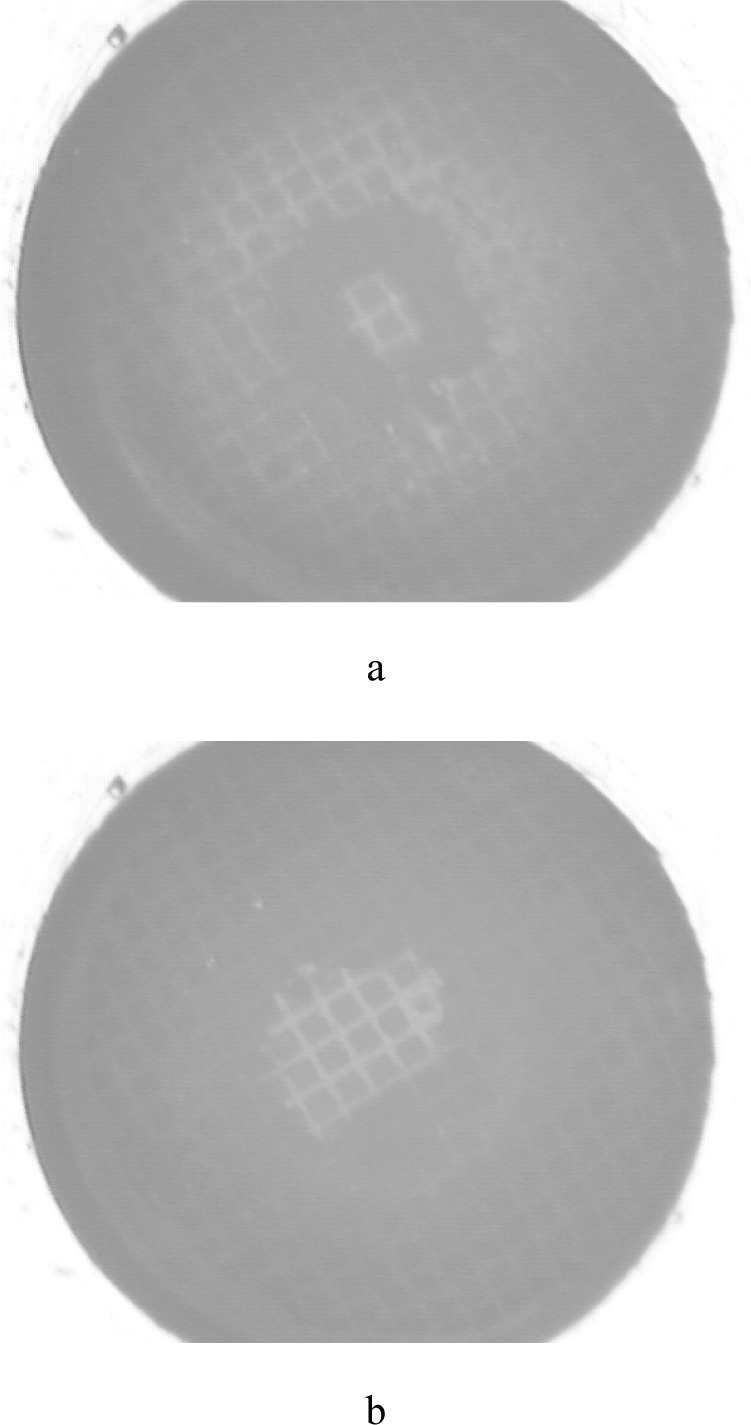
Digital images from the pole view from the latex bubble test collected at (a) 0 Pa and (b) 1.38 × 10^4^ Pa.

**Fig. 8:**
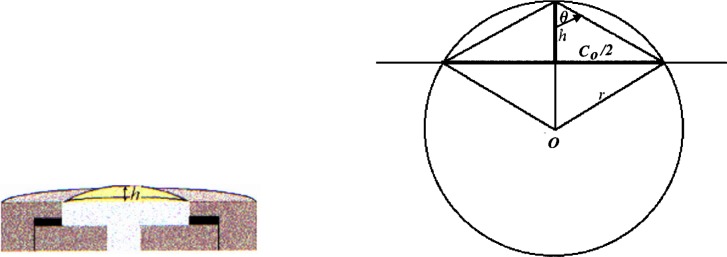
Drawings showing the origin of measurement *h* and the geometry used to solve for *r*.

To test the system, measurements were made on commercially obtained latex. A disk of latex, nominally 4 mm in diameter by 0.075 mm thick, was inserted and patterned in the biaxial fixture, and attached to the pressurizing system. All air was removed from the biaxial fixture, and as much as possible from the tubes, bellows, and pressure transducer. Because the pressures experienced by the pulmonary artery are relatively low, the test pressures will remain correspondingly low. The latex sheet was pressurized in 690 Pa increments, starting from 0 Pa up to 1.72 × 10^4^ Pa. Images were collected at each pressure increment from both cameras. Due to the isotropic behavior of latex, camera 1 collected only one image, rather than multiple images at various angles of rotation. Figure 6 shows images from camera 1 collected at 0 Pa, 6.90 × 10^3^ Pa, and 1.38 × 10^4^ Pa, illustrating the uniform inflation exhibited by the latex. Figure 7 shows images from camera 2 collected at 0 Pa and 1.38 × 10^4^ Pa. In the initial image (0 Pa) the contrast of the OsO_4_-stained pattern is poor, but upon loading the pattern becomes more distinct. The poor initial contrast made it difficult to identify points that could be traced through subsequent images to arrive at a mathematical expression for the surface for each pressure increment. For this experiment only, as the latex is isotropic and the stretch ratio is <<2, it was assumed that the deformation was spherical and could be determined from *h* (see Fig. 8). The value for *h* was obtained from the digital pictures by assigning a baseline for the suite of images where *h* = 0, then locating the interface between the latex and water, based on a change in intensity, at the point estimated to be the apex. The length *h* was measured directly from the image using a commercially available image-analysis program.

Using the geometry shown in Fig. 8, assuming the value for *C*_0_/2 to be a constant equal to 1.39 mm throughout the test, and knowing the measured value for *h*, we can calculate *r* for each pressure increment. From the relationship tan*θ* = *C*_0_/2*h*, and recognizing that the hypotenuse of that right triangle is the base of an isosceles triangle, the sides of that triangle, which are equal in length to the radius of the circle, can be computed.

## 5. Results

For calculating the equibiaxial stress in the material, we start with the Young-Laplace law for inflation of spheres:

$$T=\frac{P\cdot r}{2},$$ (3)

where *T* is the tension at the apex of the bubble, *P* is the inflation pressure, and *r* is the radius of curvature at the apex, as shown in Fig. 8. The equibiaxial stress is then:

$$\sigma =\frac{T}{\delta },$$ (4)

where *σ* is stress and *δ* is thickness. For simplicity, the material is assumed to be incompressible, which allows for the thickness to be calculated from the original thickness by use of the incompressibility condition:

$$\delta =\frac{\delta_{0}}{\lambda^{2}},$$ (5)

where *δ*_0_ is the initial, strain-free thickness (75 µm) and *λ* is the biaxial stretch,

$$\lambda =\frac{C}{C_{0}},$$ (6)

where *C* is the length of the arc of the bubble and *C*_0_ is the initial length across the sample (2.78 mm), or the diameter of the aperture of the fixture. Therefore, we measure only two variables: pressure, *P*, and the height of the bubble, *h*. From *h* and *C*_0_ we can calculate the radius of curvature of the bubble, *r*, as shown from the geometry in Fig. 8.

**Fig. 9:**
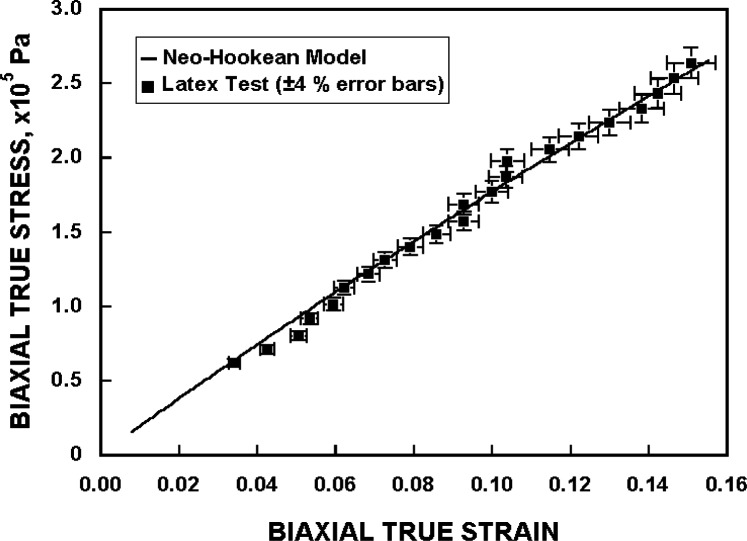
The stress-strain curve for the latex sample, compared with the theory of large elastic deformation.

The results of the measurements are shown on a plot of true stress versus true strain in Fig. 9, along with a curve showing the expected result from the theory of large elastic deformations. Using the strain energy function of Eq. (2), an equation for the biaxial true stress is derived [18] as

$$\sigma =\mu \left(\lambda^{2}-\frac{1}{\lambda^{4}}\right),$$ (7)

where *µ* is calculated to be 3.21 × 10^5^ Pa, based on a fit to the current data. The standard deviation of the biaxial true stress about the model line is 5.8 × 10^3^ Pa. The biaxial true strain is obtained from the biaxial stretch using *ε* = ln(*λ*).

The measurement data show no bias, as they are essentially randomly scattered around the theoretical curve. The low value of final strain, 13.2 %, at the highest pressure measured, 1.72 × 10^4^ Pa, is well within the elastic limit for latex [26]. This low strain value should also preclude any hysteretic effects, which may be seen at these low, and lower, pressures when testing tissue.

## 6. Analysis of Experimental Uncertainty

This measurement method is very simple in principle, and therefore it is capable of low uncertainty. While the measurement and analysis of the homogeneous latex sample differs from subsequent measurements that will be made on tissue samples, the parameters measured are very similar, so the minimization of experimental uncertainty applies to both. Both methodologies involve the measurement of only three parameters. In the case of the latex sample, measurements of *C*_0_, *h*, and *P* are required. Measurement of tissue will likewise require *P*, a displacement measurement corresponding to *h*, and a baseline length measurement corresponding to *C*_0_. For the analysis given in this paper, *C*_0_ is not actually measured, but is instead set at 2.78 mm, the size of the aperture in the fixture. However, this does not necessarily set the value at exactly 2.78 mm. Particularly at low pressures, the material may remain seated on the O-ring inside the fixture and not be contacting the aperture edge, thereby slightly decreasing the actual value of *C*_0_. A realistic and conservative value for the relative standard uncertainty (systematic) of the distance *C*_0_ is ±1.0 %. The relative standard uncertainty of the displacement measurement, *h*, is ±4.0 %, based on the pixel size of the images and the repeatability with which one typically makes this measurement. The pressure transducer and associated amplification electronics yield a relative standard uncertainty of ±0.05 % for the measurement of *P*. The uncertainties of *h* and *P* are random type uncertainties. By summing the relative standard uncertainties in quadrature, the combined standard uncertainty of the stress and strain measurements is about 4 %. This assumes that there is no uncertainty associated with the neo-Hookian model itself. Future tests on tissue will incorporate improvements, including collecting images at higher magnification and using a camera of higher resolution, which will optimize the number of pixels in the area of interest.

## 7. Conclusions

We used latex as a sample material to test the performance of a new measurement system for studying the mechanical properties of normotensive and hypertensive pulmonary arteries from rats. The test has demonstrated the high accuracy and precision that are required for testing the rat arteries. The system and the automation provided a standard uncertainty of ≈ 4 % in this measurement, and simple modifications in the future will yield even higher accuracy. The precise control and improved accuracy of the measurement should enable reliable determination of anisotropy in addition to the mechanical properties of arteries. The results of the latex test demonstrate the validity of the test system.
